# Healthcare Application of Failure Mode and Effect Analysis (FMEA): Is There Room in the Infectious Disease Setting? A Scoping Review

**DOI:** 10.3390/healthcare13010082

**Published:** 2025-01-04

**Authors:** Marco Vecchia, Paolo Sacchi, Lea Nadia Marvulli, Luca Ragazzoni, Alba Muzzi, Lorenzo Polo, Raffaele Bruno, Flavio Salio

**Affiliations:** 1 Division of Infectious Diseases Unit I, Fondazione IRCCS Policlinico San Matteo, 27100 Pavia, Italy; p.sacchi@smatteo.pv.it (P.S.); leanadia.marvulli01@universitadipavia.it (L.N.M.); raffaele.bruno@unipv.it (R.B.); 2 CRIMEDIM—Center for Research and Training in Disaster Medicine, Humanitarian Aid and Global Health, Università del Piemonte Orientale, 28100 Novara, Italy; luca.ragazzoni@med.uniupo.it; 3 Medical Direction, Fondazione IRCCS Policlinico San Matteo, 27100 Pavia, Italy; a.muzzi@smatteo.pv.it; 4 Department of Quality and Risk Management, Fondazione IRCCS Policlinico San Matteo, 27100 Pavia, Italy; l.polo@smatteo.pv.it; 5 Department of Clinical Surgical Diagnostic and Pediatric Sciences, University of Pavia, 27100 Pavia, Italy; 6 Emergency Medical Teams, Country Readiness Strengthening Department, World Health Organization, 1211 Geneva, Switzerland; saliof@who.int

**Keywords:** FMEA, risk analysis, risk management, infectious diseases, healthcare, infection control

## Abstract

**Background**: Failure mode and effect analysis (FMEA) is a valuable risk analysis tool aimed at predicting the potential failures of a system and preventing them from occurring. Since its initial use, it has also recently been applied to the healthcare setting, which has been made progressively more complex by technological developments and new challenges. Infection prevention and control (IPC) is an area that requires effective strategies. The aim of this study is to review the literature on the employment of FMEA in the healthcare environment, with special consideration for its application in the infectious disease setting. **Methods**: An extensive search was carried out in two international and public databases, PUBMED and EMBASE; we included all studies regarding the use of FMEA in hospital settings and human patient care processes. **Results**: A total of 163 studies published over the period from 2003 to 2023 were included for data extraction. These studies were analyzed regarding bibliometric data (publication year and country of origin), the healthcare issues to be addressed, the application fields, and the utilized FMEA methods. Among these, 13 studies were found that took an interest in infectious diseases. **Conclusions**: FMEA can be effectively used for healthcare risk assessment. Its implementation as a standard tool in healthcare settings, though demanding, may serve as an important tool for preventing the risk of biohazard incidents, epidemics, and environmental contamination, thereby improving safety for both patients and healthcare workers.

## 1. Introduction

Failure mode and effect analysis (FMEA) is a prospective risk analysis tool specifically designed to predict every possible failure mode of a given process, project, operating procedure, product, or service assembly line. It is also aimed at reducing the most relevant ones by proposing risk mitigation actions [1]. The technique considers three risk factors: the occurrence (O), which is the probability of failure; the severity (S) of a failure; and the detectability (D), which is the probability of not detecting failure.

The initial application of this risk analysis method lies in the military and aeronautical sectors in the United States and was implemented in the early 1940s; subsequently, it was applied in the aerospace industry in the 1960s and, finally, it was exported to the industry and production of hardware products [2,3]. After the publishing in the United States of the landmark report “To err is human: building a safer health system” in the 1990s [4], a new concept in the healthcare sector was introduced that defined FMEA as a high-risk system in which errors could result in potential harm and serious consequences for patients [5]. As the global burden of unsafe care has been progressively unmasked, it is estimated that a significant proportion of medical adverse events are preventable [6]. FMEA can be a valuable tool for the regular examination of healthcare systems.

The application of FMEA to the healthcare risk assessment has increased in recent years for two main causes. On the one hand, technologies and techniques in the medical field are undergoing constant evolution, thus adding complexity to healthcare processes. On the other hand, the epidemics of new, emerging, and re-emerging infectious pathogens that the world has witnessed in the last decades have revealed the necessity for updated and effective strategies for infection prevention and control (IPC), which are of utmost importance in the prevention of infections by multi-drug resistant organisms (MDROs), causing the so-called “silent pandemic” and one of the major threats to the public health in the 21st century. Finally, the regulatory rules, such as ISO9001 2017 and Joint Commission International, have pointed out the need for a quick evaluation and detection of risk to improve patients’ safety. In this context, it is important to understand how to apply a tool to adapt the process to this need.

In the field of infectious diseases, epidemiological risk models such as Susceptible, Infected, and Recovered (SIR) are widely used to assess the course of an epidemic.

The SIR model belongs to the family of compartmental models in epidemiology: by compartmentalising a population affected by an epidemic into Susceptible (S), Infected (I), and Recovered or Deceased (R) subjects, it enables an accurate prediction of both the number of infections in progress and their time course in order to inform public health officials and the healthcare system. This tool therefore finds extensive application in predicting the course of epidemics and allows the health authority to implement measures to respond to and mitigate the spread of an epidemic [7]. 

Although this model is reliable in analysing the risk of the spread of a contagious disease, it cannot be applied in the risk analysis of other types of healthcare processes in infectious diseases, such as infection control, donning and doffing processes with personal protective equipment (PPE), or in the prevention of healthcare-associated infections (HAIs).

The aim of this article is to review the relevant literature on the use of FMEA in a healthcare environment with special consideration for its application in an infectious disease setting.

## 2. Materials and Methods

This study follows the Preferred Reporting Items for Systematic Reviews and Meta-Analyses extension for scoping reviews (PRISMA-ScR) guidelines [8]. A review protocol was entered into the Open Science Framework database (Registration DOI: https://doi.org/10.17605/OSF.IO/PR4QH).

### 2.1. Data Sources and Management

We searched two international and public databases, PUBMED and EMBASE, using predefined search strings on 31 October 2023 (available at Supplementary Table S1). The research was carried out with the help of the librarian of the Foundation IRCCS Policlinico San Matteo.

Abstract and full-text reviews were performed independently by two project team members. Reviewers resolved disagreements by discussion and, when necessary, through adjudication by a third reviewer.

### 2.2. Inclusion and Exclusion Criteria

We included all studies regarding the use of FMEA in hospital settings or human patient care processes that were written in English or Italian and published in international peer-reviewed journals between 1995 and 2023. We decided to start collecting research from 1995 as the application of FMEA in healthcare settings dates back to the 1990s and we wanted to cover almost thirty years of literature. We excluded grey literature like unpublished articles, conference abstracts, and any non-peer-reviewed works, as well as studies in languages other than English and Italian, studies published before 1995, studies that covered the use of other risk analysis methods in the healthcare setting, and studies considering other risk analysis tools or applications of FMEA in out-of-hospital settings.

### 2.3. Data Extraction

For each study included, data were extracted by M.V. and L.M.N. to a dedicated spreadsheet. Data of interest were the healthcare issues to be solved (classified according to Hu-Chen Liu et al. [9]), the application area, the study design, the number of risks identified, and whether suggestions or interventions adopted to resolve or mitigate the risk were provided.

### 2.4. Risk of Bias

Given the heterogeneity of the studies included in this review, bias was assessed qualitatively using Joanna Briggs Institute’s critical appraisal checklists [10]. Quality was considered adequate if all the items in the checklist were marked “yes”, but studies with inadequate quality were not discarded in order to collect all available data. Quality assessment was performed independently by M.V. and P.S.

### 2.5. Data Synthesis and Analysis

We quantitatively and qualitatively summarized and analyzed the characteristics of the articles included using summary tables and graphs.

## 3. Results

**Figure 1:**
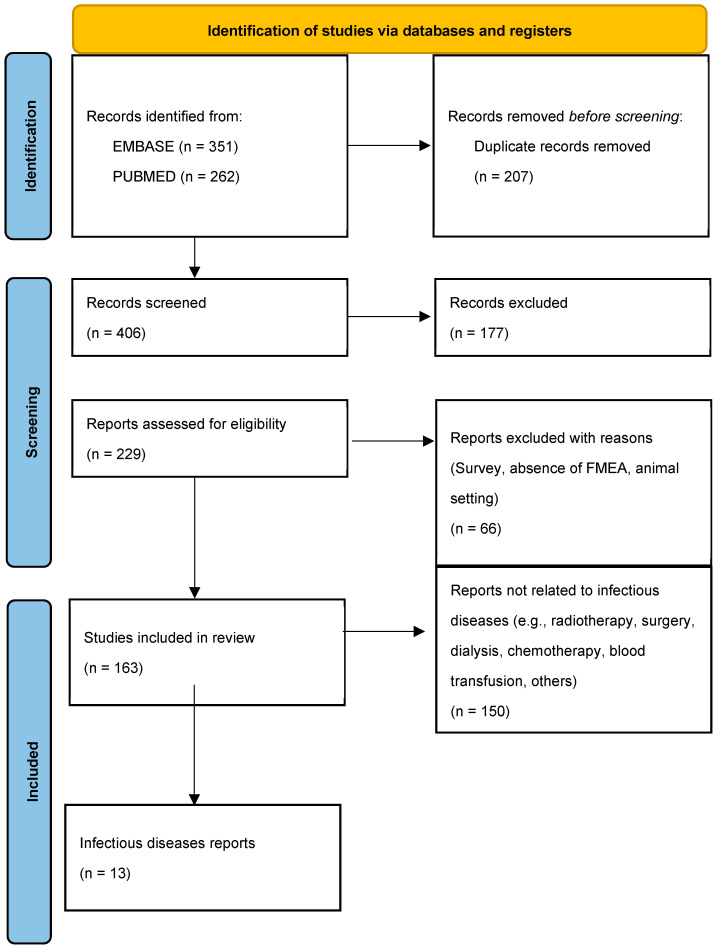
PRISMA flowchart for study identification, screening, and inclusion.

After a literature search in the two databases indicated, 613 eligible published studies were identified. Of these, 207 of these were duplicates that were removed. The titles and abstracts of 406 records were manually screened for full-text assessment. Overall, 229 articles were selected for full-text evaluation. Reasons for exclusion included the presence of only expert surveys, the absence of a real application of FMEA analysis, and the application of FMEA in the animal sector. A total of 163 studies were included for data extraction. Citation and study characteristics included in the application areas are shown in extenso in Supplementary Table S2. The PRISMA flowchart summarizing all the steps performed for study identification, screening, and inclusion is reported in Figure 1.

### 3.1. Studies’ Characteristics

**Figure 2:**
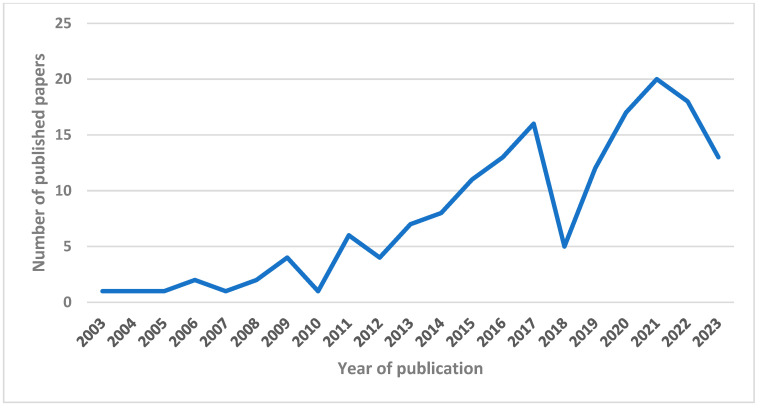
Line chart showing the trend of publication.

The 163 studies included in this review were published between 2003 and 2023 (Figure 2). Although the number of publications on the use of FMEA in healthcare is increasing, the years 2018, 2022, and 2023 showed deflections compared to previous years. This trend detected in our work is representative of the global trend of publications detected using the search strings provided.

**Figure 3:**
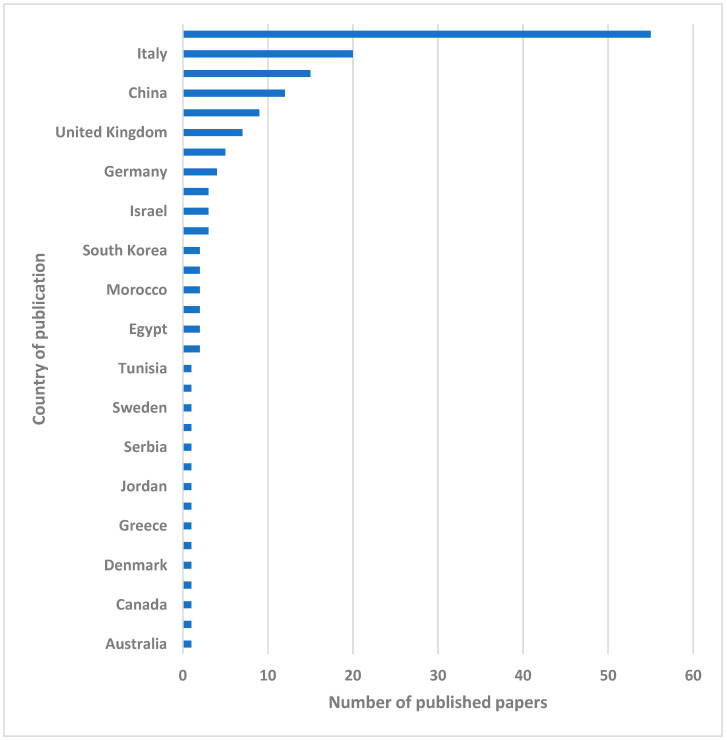
Bar chart showing the numbers of published papers divided by nationality.

The selected studies came from 32 different countries, with the major contribution coming from the USA (55 articles), followed by Italy (20 articles), Spain (15 articles), and China (12 articles) (Figure 3).

**Figure 4:**
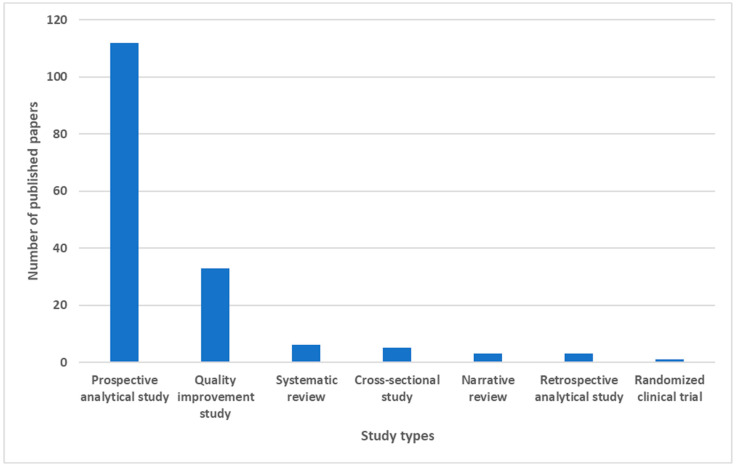
Histogram showing the numbers of published articles divided by study type.

Among the 163 studies analyzed, the two most represented study types were prospective analytical studies (n = 112, 68.71%) and quality improvement studies (n = 33, 20.25%); from these two categories, we obtained most of the applications of FMEA in the health sector. The remaining studies were distributed among systematic reviews (n = 6, 3.68%), cross-sectional studies (n = 6, 3.68%), narrative reviews (n = 3, 1.84%), retrospective analytical studies (3, 1.84%), and randomized clinical trial (n = 1, 0.61%). Figure 4 shows the distribution of the published papers according to the study types.

Among the four categories of application, the most popular was healthcare processes (n = 129, 79.14%), followed by hospital management (n = 20, 12.27%), medical equipment/production (n = 6, 3.68%), and hospital informatization (n = 4, 2.45%). In four studies (2.45%), FMEA was applied to two categories.

In the two most represented categories, sub-categories of application could be recognized. In the healthcare process category, the majority of studies reported the use of FMEA in the radiation therapy field (46 out of 129, 35.66%): 12 studies applied FMEA as a general risk analysis tool in a radiation oncology department, while other studies used FMEA to implement new techniques (e.g., scanned proton beam radiotherapy, intensity-modulated radiation therapy, external beam radiotherapy, imaging-guided radiotherapy, and stereotactic radiation surgery) or to specific treatments (e.g., breast, lung, ocular, gynaecology, prostate, liver, and skin radiotherapy). Twenty studies (15.50%) fell under the sub-category of “other treatment processes”, which included a variety of healthcare processes, the most represented of which was dialysis (3 studies). Thirteen studies (10.08%) applied FMEA to some healthcare support activities, such as the delivery of surgical instruments and patient safety in paediatric emergency care. Other sub-categories were medication use processes (n = 11, 8.53%), chemotherapy (n = 9, 6.98%), blood transfusion and testing in clinical laboratories (n = 7, 5.43%), surgery (n = 5, 3.88%), and ICU processes (n = 4, 3.10%).

In the hospital management category, two-thirds of studies were almost equally distributed among the sub-categories of healthcare support management (7 out of 20, 35.00%) and communication and patient handoff (6 out of 20, 30.00%).

### 3.2. Failure Modes and Corrective Actions

The majority of the articles (n = 153, 93.87%) listed specific failure modes. Overall, 9564 failure modes were identified; the mean number of failure modes identified per study was 62.51, with the 25th and 75th percentiles of the data being equal to 17 and 87, respectively. The minimum number of identified failure modes in a single study was one, whereas the study that highlighted the maximum number of failure modes listed 388 failure modes.

Out of 163 articles, 122 of them (74.85%) proposed and listed specific and clear corrective actions.

### 3.3. Application of FMEA to Infectious Diseases

Looking through the four categories, FMEA was applied to the field of infectious diseases, antimicrobials, and infection control in 13 out of the 163 articles analysed (7.98%).

The main characteristics of these articles are summarized in Table 1. The detailed review is presented in Appendix A.

The infectious disease setting involves not only healthcare processes but also the prevention of the spread of microorganisms and the protection of healthcare workers. Among the thirteen analyzed articles, eight have prospective analytical design, four were cross-sectional studies and one was a quality improvement design. The categories examined were healthcare processes (7 studies), hospital management (5 studies), or both (1 study). The application area ranged from drug prescription [13,18,21], prevention of hospital outbreak or spread of MDRO [14,17,19,20,22,23], detection of medical errors or failure of healthcare processes in ED or in ICU [11,12], use of PPE [15], and waste management [16]. Some failure modes were identified, such as poor standard procedure application in prevention processes, latency in response phase in ED and ICU, wrong PPE doffing, and waste disposal. In particular, Islam et al. applied FMEA to manage both infection control and radiotherapy risk during infectious disease outbreaks, such as the SARS-CoV-2 pandemic [17]. This study introduced a new subclass of failure modes known as infection control failure modes (ICFM), which were defined as any instance when the transmission of infection occurred within the clinic.

## 4. Discussion

This scoping review aims to provide a general overview of the application of FMEA in healthcare settings. To this end, 163 articles relevant to the scope of the work were identified. Most of them consisted of prospective analytical studies and quality improvement studies. It is interesting to note that, in the analyzed period, the number of publications with this topic increased over time, with a peak occurring during the pandemic year.

Recent advances in medicine due in part to the availability of new technologies have led not only to improvements in the quality of patient care but also to the challenge of improving patient safety. In fact, the last few years have seen an increase in the number of studies on the risks associated with treatment processes because of the identification of new methods for process failure and risk reconnaissance, particularly with regard to the use of diagnostic and therapeutic tools and the use of drugs, especially in oncology, that were previously unavailable.

The COVID-19 pandemic has posed serious challenges for the delivery of quality care in healthcare systems. For example, in India, one of the countries that was hit the hardest by the COVID-19 pandemic, risk analysis models have been applied to contain the spread of the virus in hospital settings in order to balance the care needs of patients with the safety of healthcare workers [24,25,26]. Thus, it is not surprising that it has coincided with a reconfirmed interest in FMEA.

Applications of FMEA appear varied, with a clear predominance of the category of healthcare processes. The most common healthcare risk analysis problem is related to radiation therapy, given the high potential of severe errors due to the use of radioisotopes, both in terms of the dose administered to patients and the environmental contamination with radioactive materials. In the last decade, after some serious radiation therapy accidents, incident learning systems have been recognized as a valid tool to prevent adverse events [27]. Similarly, the administration of chemotherapeutics is a healthcare area in which FMEA is applied extensively to ensure the safety of infusions, reduce the risk of contamination of preparations and infusion lines, and optimize pharmacological prescriptions. Cancer treatments are a common situation in which fatal medication errors occur, according to different reports [28,29,30]. Blood transfusions are another important area of application, given that errors at every step of the blood chain can lead to fatal mistranfusions. Electronic patient identification systems have been proven to significantly reduce the risks associated with mislabeled samples [31]. Surgical, administrative, computerization, and diagnostic areas do not see extensive use of the FMEA technique in their respective analyses of risks, even if they are present and potentially life threatening.

Regarding the field of infectious diseases, 13 studies have been identified. In the majority of them, FMEA was applied to the area of infection prevention and control, including hand hygiene and the use of personal protective equipment, in order to reduce the risk of hospital outbreaks and prevent hospital-acquired infections, especially those caused by MDROs. As shown in the most recent data [32], healthcare-associated infections represent a major public health problem, leading to significant morbidity and mortality, higher healthcare costs, litigations, and an increased use of antimicrobials. In recent years, efforts aimed at preventing healthcare-associated infections have continued to grow at a national level, with some successful examples [33]. The promising results shown in the studies analyzed here may pave the way for an active role of risk management tools such as FMEA in the prevention of hospital outbreaks by MDROs and in antimicrobial stewardship strategies. Specifically, a study conducted in the ICU setting showed that FMEA could effectively reduce the incidence of catheter-related bloodstream infections, which could be prevented by adequate staff training [34,35]. As the use of biomedical devices, especially those implanted, continues to increase, this positive result suggests a novel approach to the problem of implant-associated infections.

The results of this paper emphasize that FMEA application in the planning phase of healthcare pathways can be a useful tool in finding and resolving potential failure modes. In the infectious disease setting, many failure modes can be identified, ranging from the use of personal protective equipment to contact precaution, with each one being a potential risk for patients and healthcare operators.

The study presents several limitations. The most relevant one is related to the wide heterogeneity of FMEA’s application areas; however, only in some of them is extensive application found, such as in radiotherapy, blood transfusion, and drug administration. This finding determines the high variability of the results obtained. Thus, it is difficult to compare the results. Another limitation lies in the different typologies of articles selected (prospective analytical studies, quality improvement, review, etc.), which produces a confounding factor on the results, the number of RPNs identified, the areas of application, and the presence or absence of suggested corrective actions. In addition, approximately 25% of the published papers do not have clearly explained corrective actions, thus reducing the possibility of analyzing each article extensively.

## 5. Conclusions

This review sheds light on the recent use of FMEA for healthcare risk assessment. Specifically, 13 papers regarding infectious diseases, antimicrobials, and outbreak prevention settings have been identified. The results allow us to answer our initial question affirmatively, recognizing an important application for FMEA in the field of infectious diseases, particularly regarding the use of personal protective equipment, hospital infection control, outbreak prevention, infectious medical waste management, prevention of catheter-related infection in intensive care units, and sepsis management in emergency departments. Given the recent SARS-CoV-2 pandemic and the relative increase in recent years of pandemic and epidemic incidence around the globe, it is crucial that healthcare workers, hospital managers, and local and government authorities become familiar with this risk analysis model. If applied to the biosafety field, it may serve as an important tool for preventing and mitigating the risks of biohazard incidents, epidemics, and environmental contamination. Nevertheless, the implementation of FMEA as a standard tool in healthcare settings requires multi-level commitment, training, reliable process analysis systems, reporting, and verification of proposed corrective actions.

The results of the review show that as the complexity of a process increases, so does the risk, necessitating a well-performed risk assessment before and during the implementation of this process. FMEA, which breaks down the process into stages and allows them to be analyzed separately, is a well-suited tool to evaluate different types of processes, enabling the identification of failure modes and providing risk mitigation strategies. In the infectious disease context, prevention is crucial at several levels, ranging from the safety of healthcare workers and patients to outbreaks in both hospital and community settings; thus, these situations need a risk analysis tool to ensure the best outcome in public health management.

## Figures and Tables

**Table 1:** Application of failure mode and effect analysis (FMEA) in infectious diseases (ED = Emergency Department; ICU = Intensive Care Unit; MDRO = Multi Drug-Resistant Organisms; OPAT: Outpatient Parenteral Antimicrobial Therapy; RPN: Risk Priority Number; and USA: United States of America).

Authors	Year of Publication	Country	Type of Study	Category	Application Area	Number of Failure Modes	RPN	Suggested Corrective Actions
Alamry A. et al. [11]	2014	Saudi Arabia	Prospective analytical study	Hospital management	Treatment of septic patients from ED	48	Yes	Yes
Alimohammadzadeh K. et al. [12]	2017	Iran	Cross-sectional study	Hospital management; Healthcare process	Common clinical errors in neonatal ICU	57	Yes	Yes
Daniels L.M. et al. [13]	2015	USA	Prospective analytical study	Healthcare process	Antimicrobial drugs interaction with anticoagulants	134	Yes	Yes
Davis N.R. et al. [14]	2020	USA	Prospective analytical study	Hospital management	Simulation-based clinical systems testing in pandemic response	109	No	Yes
Gurses A.P. et al. [15]	2019	USA	Prospective analytical study	Healthcare process	Doffing-enhanced personal protective equipment	103	Yes	Yes
Ho C.C. et al. [16]	2011	Taiwan	Prospective analytical study	Hospital management	Infectious medical waste management	19	Yes	Yes
Islam N.M. et al. [17]	2023	USA	Prospective analytical study	Healthcare process	Infectious disease outbreak in radiotherapy	90	Yes	No
Latt E.E.V. et al. [18]	2021	Morocco	Prospective analytical study	Healthcare process	Drug prescription and administration during SARS-CoV-2 pandemic	12	Yes	Yes
Li X. et al. [19]	2017	China	Prospective analytical study	Healthcare process	Prevention of catheter-related infection in ICU	25	Yes	Yes
Lin L. et al. [20]	2021	China	Quality improvement study	Healthcare process	Prevention of MDRO infections in ICU	5	Yes	Yes
Sadler E.D. et al. [21]	2021	USA	Prospective analytical study	Healthcare process	OPAT	13 + 10	No	Yes
Teklewold B. et al. [22]	2021	Ethiopia	Cross-sectional study	Hospital management	Prevention of SARS-CoV-2 transmission in the ED	22	Yes	Yes
Xu Y. et al. [23]	2021	China	Prospective analytical study	Hospital management	Prevention of MDRO infections in oral and maxillofacial surgery	7	Yes	Yes

## Data Availability

No new data were created in this study. Template data collection forms, data extracted from included studies, data used for all analyses, and analytic code are available upon reasonable request.

## Supplementary Materials

The following supporting information can be downloaded at https://www.mdpi.com/article/10.3390/healthcare13010082/s1: Table S1—Search queries and Table S2—List of all the selected articles on the use of FMEA in healthcare setting [9,11,12,13,14,15,16,17,18,19,20,21,22,23,36,37,38,39,40,41,42,43,44,45,46,47,48,49,50,51,52,53,54,55,56,57,58,59,60,61,62,63,64,65,66,67,68,69,70,71,72,73,74,75,76,77,78,79,80,81,82,83,84,85,86,87,88,89,90,91,92,93,94,95,96,97,98,99,100,101,102,103,104,105,106,107,108,109,110,111,112,113,114,115,116,117,118,119,120,121,122,123,124,125,126,127,128,129,130,131,132,133,134,135,136,137,138,139,140,141,142,143,144,145,146,147,148,149,150,151,152,153,154,155,156,157,158,159,160,161,162,163,164,165,166,167,168,169,170,171,172,173,174,175,176,177,178,179,180,181,182,183,184]. Studies related to infectious diseases, antimicrobials and infection control are highlighted in bold type (ADHD: attention-deficit/hyperactivity disorder; CBC: complete blood count; CIEDs: cardiac-implanted electronic devices; ED: emergency department; ICU: intensive care unit; IVF: in vitro fertilization; MDROs: multi-drug resistant microorganisms; MRI: magnetic resonance imaging; N/A: not applicable; OPAT: outpatient parenteral antimicrobial therapy; and USA: United States of America). Supplementary file: Preferred Reporting Items for Systematic reviews and Meta-Analyses Extension for Scoping Reviews (PRISMA-ScR) Checklist.

## Appendix A. Application of Failure Mode and Effect Analysis (FMEA) in Infectious Diseases

In the first study, Alamry et al. applied FMEA to the treatment of septic patients in the ED and recorded processes under three major phases (recognition of severe sepsis, referral, and resuscitation) [11]. In this prospective analytical study, a total of 48 failure modes with a mean RPN of 270 were identified. All critical processes were related to the recognition and resuscitation phases. Among the risk-reduction suggestions, the introduction of an electronic alert system was proposed, as well as a weekly reminder for the physicians regarding the core elements of the sepsis bundle.

In the second study, Alimohammadzadeh et al. conducted a cross-sectional study in a neonatal ICU to assess common medical errors in four key processes, including drug administration, infection control, medical equipment uses, and laboratory tests [12]. Of the 27 total high-risk failure modes (RPN > 65), 12 were detected in the infection control process: the highest RPNs were related to improper and incomplete washing and disinfecting of hands (RPN = 127), non-compliance with the hand washing principles in five situations (RPN = 126), and non-compliance with the sterilization principles in catheterization, chest tube, intubation, and bladder catheterization (RPN = 104). The suggestions offered were aimed at increasing the detectability and reducing both the occurrence and severity of impact.

In the third study, Daniels et al. conducted a quality-improvement project to analyze the processes leading to an increased risk of elevated INR levels in warfarin-treated patients receiving concomitant antimicrobial therapy, namely, trimethoprim–sulfamethoxazole, metronidazole, fluconazole, miconazole, or voriconazole [13]. The FMEA analysis identified 134 failure modes; the four highest-risk failure modes regarded the lack of identification of all patients taking warfarin by electronic medical records and the unawareness of the patients taking the drug therapy by the antimicrobial and anticoagulant prescribers. The quantitative impact of each proposed intervention was estimated through an effort–benefit analysis, which led to the identification of the four most effective interventions.

In the fourth study, Davis et al. implemented FMEA in a simulation-based clinical systems test in order to redeploy mobile pediatric emergency response teams as alternative care sites during surge events, such as pandemics [14]. This process allowed the rapid identification of 109 failure modes (here defined as latent safety threats) regarding resource, system, facility, and clinical performance issues. Suggested corrections included additional staffing, ease of access to commonly used supplies, structural modifications, and proper registration methods.

In the fifth prospective analytical study, Gurses et al. described 103 possible failure modes during the use of doffing-enhanced personal protective equipment in a special pathogens treatment center [15]. The failure modes associated with higher RPN were associated with staff moving between clean and contaminated areas, glove removal, apron removal, and self-inspection while preparing to doff. Risk mitigation strategies included providing visual indications of contaminated and contamination-free areas of doffing rooms, providing education and training in the understanding of germ theory in disinfection, and identifying and assessing risks. Other strategies included spatial orientation awareness between team members, the environment, and equipment and the use of assertiveness, assistance, back-up behaviors, and cross-checking between all team members.

In the sixth study, Ho et al. [16] wanted to evaluate the potential risk causes in the process of infectious medical waste disposal, including sharp implements. Nineteen failure modes were identified, and six of them were ranked as priority items for improvement. Although not being the failure mode with the highest RPN, erroneous discarding of infectious waste by medical staff was believed to be one of the more urgent areas for improvement because of the domino effect on other failure modes. Among the proposed interventions, increasing education and training about waste classification for medical staff, patients and their families, and cleaning staff was highlighted.

In the seventh study, Islam et al. applied FMEA to manage both infection control and radiotherapy risks during infectious disease outbreaks, such as the SARS-CoV-2 pandemic [17]. The initial assumption was that the application of infection control measures may alter the standard clinical process flow, thus creating new failure modes or increasing the occurrence of existing ones. This study introduced a new subclass of failure modes known as infection control failure modes (ICFM), which were defined as any instance when transmission of the infection occurred within the clinic. In this case, the infection RPN was calculated by considering the duration of exposure to a potential infection source, the distance from the potential source, and the degree of gear protection.

In the eight study, Latt et al. evaluated the risk of transmission of SARS-CoV-2 and infection during the process of outpatient drug dispensation [18]. Twelve failure modes were highlighted, ten of which were considered critical and were mostly related to the patient reception phase. Corrective measures included distancing, early detection of symptoms, the requirement of wearing protective equipment, the reminder to both patients and personnel of the rules of hand hygiene, and the respect of the procedures of the dispensation to guarantee asepsis and safety.

In the ninth study, Li et al. wanted to reduce the incidence of catheter-related bloodstream infections in ICU [19]. The FMEA analysis identified 25 failure modes; those with RPN > 100 were selected as priority items for corrective action implementation, involving the nursing care system. Statistical analysis showed that the average infection rate in the observation group was significantly lower than in the control group (from 5.19% to 1.42%). Mitigation strategies include choosing the right catheter for the vein, strictly carrying out aseptic operation, choosing the healthcare operators’ formation, choosing the most appropriate materials, choosing good air-permeable gauze, choosing the aseptic, managing correct intravenous lines, performing timely flushing and locking, cohorting the patients, and isolating and properly using protective personal equipment.

In this tenth quality improvement study, Lin et al. described how the use of FMEA in ICU can effectively reduce multi-drug-resistant bacteria outbreaks in ICU patients [20]. Two groups of ICU patients were identified: the control-group patients received conventional ICU care and the observation group was treated by FMEA. Five failure modes were identified. The RPN value of each one was significantly lower in the observation group compared to the control, as was MDR bacteria incidence and mortality rate of patients with MDROs. Additionally, the satisfaction level of the observation group with the ICU treatment was higher. Mitigation strategies include contact isolation measure implementation. A closed disposable sputum suction tube is used to carry out sputum suction operation, implementation of aseptic procedures, local skin disinfection with bactericidal drugs before invasive operation, oral cleaning care for patients with orotracheal intubation, proper cleaning of ventilators, maintaining hand hygiene standards, bed unit cleaning and disinfection, frequent opening of department windows, and implementation of antimicrobial stewardship programs.

In the eleventh study, Sadler et al. described the application of FMEA to improve transitions of care in patients discharged on outpatient parenteral antimicrobial therapy [21]. Twenty-three failure modes were identified and classified into five areas of focus: OPAT electronic order set, critical tasks to be performed before patient discharge, patient education, patient follow-up and laboratory monitoring, and skilled nursing facility (SNF) communication. Suggested interventions included the update of the electronic order set, the creation of an inpatient electronic discharge checklist, the development of patient education programs, the implementation of a central OPAT outpatient database, and the creation of a pharmacist on-call pager for SNFs.

In the twelfth analyzed study by Teklewold et al., FMEA was applied to reduce the admission of asymptomatic COVID-19 patients in ED [22]. In total, 22 failure modes were identified in the patient flow process, and they were associated with the failure or lack of application of standard transmission-based precautions. Twenty-three improvement actions were proposed in the paper; they included training and compliance monitoring of COVID-19 with checklists, the availability of precaution facilities with backup, staff education, the control of the validity of thermo-scanners, the implementation of screening programs, the capacity increase of the pre-triage waiting area, proper disinfection monitoring, and the increase in the number of healthcare operators, among others.

In the thirteenth and last study, Xu et al. applied the FMEA technique to prevent multi-drug resistant organism infections in oral and maxillofacial surgery [23]. After the implementation of preventative and control measures, MDRO incidence decreased, the mastery of preventative and control knowledge among healthcare staff increased, and oral self-care ability compliance among patients increased. Additionally, a statistically significant reduction in RPN of 86.99% was observed. The risk reduction strategies adopted were the strengthening of the awareness of the sterility of medical and nursing staff and the frequency of disinfection, refining the operating procedures and environmental cleaning order, disinfection of infusion pumps, monitoring blood pressure monitors and other medical equipment, hand hygiene training or lectures for both staff and patients, education on MDRO, placing hand disinfection items at the patient’s bedside, establishing early warning mechanisms for drug resistance, and drug-resistance recording, among others.
