# Supplementary material for: Healthcare Application of Failure Mode and Effect Analysis (FMEA): Is There Room in the Infectious Disease Setting? A Scoping Review
Source: Healthcare (Basel). 2025 Jan 4;13(1):82. doi: 10.3390/healthcare13010082 (PMC11719677; doi:10.3390/healthcare13010082)
Supplement: Supplementary file 1 [file healthcare-13-00082-s001.zip › healthcare-3333370-Supplementary Tables.pdf]

Supplementary Table S1 - Search queries

Date	Database	Search query	Results
31/10/2023	PUBMED	("healthcare failure mode and effect analysis"[MeSH Terms] OR "Risk Assessment"[Mesh]) AND ("Failure mode and effects analysis" [Title/Abstract] OR FMEA[Title/Abstract]) Filters: English, Italian Sort by: Most Recent	262
31/10/2023	EMBASE	('healthcare failure mode and effect analysis'/exp OR 'risk assessment'/exp) AND ('failure mode and effects analysis'/exp OR 'failure modes and effects analysis:ab,ti' OR 'fault mode and effect analysis':ab,ti OR 'fmea':ab,ti OR 'failure mode and effects analysis':ab,ti) AND ([article]/lim OR [article in press]/lim OR [review]/lim) AND ([english]/lim OR [italian]/lim) AND [humans]/lim	351

Supplementary Table S2 - List of all the selected articles on the use of FMEA in healthcare setting. Studies related to infectious diseases, antimicrobials and infection control are highlighted in bold type (ADHD: attention-deficit/hyperactivity disorder; CBC: complete blood count; CIEDs: cardiac-implanted electronic devices; ED: emergency department; ICU: intensive care unit; IVF: in vitro fertilization; MDROs: multi-drug resistant microorganisms; MRI: magnetic resonance imaging; N/A: not applicable; OPAT: outpatient parenteral antimicrobial therapy; USA: United States of America).

Study reference	Year	Country	Type of Study	Category	Sub-category	Detailed application area	Number of failure modes	Suggested corrective actions
[1]	2019	China	Systematic review	Hospital management Healthcare process	Healthcare process	Healthcare process	N/A	N/A
[2]	2022	Tunisia	Prospective analytical study	Healthcare process	Medication use process	Medication use process	24	Yes
[3]	2013	United Kingdom	Prospective analytical study	Healthcare process	Surgery	Robotic surgery	51	Yes
[4]	2021	USA	Prospective analytical study	Healthcare process	Radiation therapy	Total body irradiation	87	Yes
[5]	2015	Egypt	Prospective analytical study	Healthcare process	Medication use process	Infusion therapy in ICU	11	Yes
**[6]**	**2014**	**Saudi Arabia**	**Prospective analytical study**	**Hospital management**	**Communication and patient handoff**	**Treatment of septic patients from ED**	**48**	**Yes**
**[7]**	**2017**	**Iran**	**Cross-sectional study**	**Hospital management Healthcare process**	**Medication administration. Testing in clinical laboratory. Infectious diseases, antimicrobials and infection control.**	**Common clinical errors in neonatal ICU**	**57**	**Yes**
[8]	2021	Brazil	Prospective analytical study	Healthcare process	Other treatment processes	Organ donation-transplantation process	30	No
[9]	2020	Egypt	Cross-sectional study	Healthcare process	Blood transfusion	Blood transfusion	38	Yes
[10]	2020	Sri Lanka	Systematic review	Hospital management Healthcare process	Medication administration. Medication use process	Medication safety	N/A	N/A
[11]	2017	Spain	Prospective analytical study	Healthcare process	Medication use process	Use of oral syringes	5	Yes
[12]	2017	Spain	Retrospective analytical study	Healthcare process	Other treatment processes	Dialysis	21	Yes
[13]	2014	Spain	Retrospective analytical study	Healthcare process	Other treatment processes	Neonatal parenteral nutrition	82	Yes
[14]	2012	Iran	Prospective analytical study	Healthcare process	ICU process	ICU process	48	Yes

[15]	2017	Iran	Systematic review	Hospital management Healthcare process	Medication administration. Medication use process	Medication administration. Medication use process	N/A	N/A
[16]	2011	United Kingdom	Prospective analytical study	Healthcare process	Chemotherapy	Chemotherapy administration	30	Yes
[17]	2020	Germany	Prospective analytical study	Healthcare process	Radiation therapy	Radiation oncology	29	No
[18]	2013	Italy	Prospective analytical study	Hospital management	Communication and patient handoff	Communication failures among health professionals working in the ED	22	Yes
[19]	2023	Morocco	Prospective analytical study	Healthcare process	Medication use process	Cytotoxics preparation	12	Yes
[20]	2014	Italy	Prospective analytical study	Healthcare process	Other treatment processes	Dialysis	92	Yes
[21]	2021	USA	Prospective analytical study	Healthcare process	Radiation therapy	Breast radiotherapy	57	Yes
[22]	2023	Italy	Prospective analytical study	Healthcare process	Chemotherapy	Chemotherapy administration	22	Yes
[23]	2019	Italy	Prospective analytical study	Healthcare process	Other treatment processes	Testicular sperm extraction	19	Yes
[24]	2013	Italy	Prospective analytical study	Healthcare process	Radiation therapy	Scanned proton beam radiotherapy	44	Yes
[25]	2023	Italy	Prospective analytical study	Healthcare process	Healthcare support activities	Colorectal cancer screening	23	Yes
[26]	2009	Italy	Systematic review	Healthcare process	Testing in clinical laboratory	Blood cross-matching, clinical chemistry analytes, and point-of-care testing	N/A	N/A
[27]	2023	Spain	Cross-sectional study	Healthcare process	Surgery	Surgery	26	No
**[28]**	**2015**	**USA**	**Prospective analytical study**	**Healthcare process**	**Infectious diseases, antimicrobials and infection control**	**Antimicrobial drugs interaction with anticoagulants**	**134**	**Yes**
**[29]**	**2020**	**USA**	**Prospective analytical study**	**Hospital management**	**Healthcare support management**	**Simulation-based clinical systems testing in pandemic response**	**109**	**Yes**
[30]	2007	USA	Prospective analytical study	Hospital management	Healthcare support management	Patient registration process	8	Yes
[31]	2006	USA	Prospective analytical study	Healthcare process	Other treatment processes	Dialysis	6	Yes
[32]	2015	Iran	Cross-sectional study	Healthcare process	Blood transfusion	Blood transfusion in pediatric emergency	77	Yes

[33]	2012	USA	Prospective analytical study	Healthcare process	Radiation therapy	Radiation oncology	108	Yes
[34]	2023	Bahrain	Prospective analytical study	Hospital informatization	None	Medication delivery	9	Yes
[35]	2017	USA	Prospective analytical study	Healthcare process	Radiation therapy	Intensity modulated radiation therapy	11	No
[36]	2021	USA	Prospective analytical study	Healthcare process	Chemotherapy	Chemotherapy preparation	16	Yes
[37]	2016	Croatia	Prospective analytical study	Healthcare process	Testing in clinical laboratory	Preanalytical process	5	Yes
[38]	2009	USA	Prospective analytical study	Healthcare process	Radiation therapy	Radiation oncology	127	Yes
[39]	2018	Australia	Prospective analytical study	Healthcare process	Radiation therapy	Radiation oncology	83	Yes
[40]	2010	USA	Prospective analytical study	Healthcare process	Surgery	Laparoscopic surgery	35+	No
[41]	2016	Italy	Systematic review	Healthcare process	Radiation therapy	Radiation oncology	N/A	N/A
[42]	2021	United Kingdom	Prospective analytical study	Healthcare process	Radiation therapy	Lung radiotherapy	36	Yes
[43]	2023	USA	Prospective analytical study	Healthcare process	Radiation therapy	Total body irradiation	128	Yes
[44]	2016	The Netherlands	Prospective analytical study	Healthcare process	Healthcare support activities	Delivery of surgical instruments	74	Yes
[45]	2016	The Netherlands	Prospective analytical study	Healthcare process	Healthcare support activities	Delivery of surgical instruments	172+158	Yes
[46]	2020	Spain	Prospective analytical study	Healthcare process	Testing in clinical laboratory	Analytical performance	10+6	Yes
**[47]**	**2019**	**USA**	**Prospective analytical study**	**Healthcare process**	**Healthcare support activities**	**Doffing enhanced personal protective equipment**	**103**	**Yes**
[48]	2021	USA	Prospective analytical study	Medical equipment/production	None	Infusion pump	17	Yes
[49]	2013	South Korea	Cross-sectional study	Healthcare process	Blood transfusion	Blood transfusion	4 to 22	Yes
[50]	2021	Jordan	Prospective analytical study	Healthcare process	Testing in clinical laboratory	Blood sampling	29	Yes
[51]	2023	China	Retrospective analytical study	Healthcare process	Other treatment processes	Emergency endoscopy esophagogastric variceal bleeding	18	Yes
**[52]**	**2011**	**Taiwan**	**Prospective analytical study**	**Hospital management**	**Healthcare support management**	**Medical waste management**	**19**	**Yes**
[53]	2022	Taiwan	Prospective analytical study	Healthcare process	Radiation therapy	External beam radiotherapy	201	No

[54]	2017	Spain	Prospective analytical study	Healthcare process	Other treatment processes	Propofol sedation in digestive endoscopy	47	Yes
[55]	2008	USA	Prospective analytical study	Healthcare process	Radiation therapy	Radiation oncology	4	No
**[56]**	**2023**	**USA**	**Prospective analytical study**	**Healthcare process**	**Infectious diseases, antimicrobials and infection control**	**Infectious diseases outbreak in Radiotherapy**	**90**	**No**
[57]	2021	Iran	Prospective analytical study	Healthcare process	Medication use process	Medication use process	10	No
[58]	2015	USA	Prospective analytical study	Healthcare process	Radiation therapy	Tomotherapy-based radiation therapy	72	Yes
[59]	2019	USA	Prospective analytical study	Healthcare process	Radiation therapy	Radiation planning assistant	68	Yes
[60]	2021	Germany	Prospective analytical study	Healthcare process	Radiation therapy	Magnetic resonance-guided radiotherapy	89	Yes
[61]	2020	Israel	Prospective analytical study	Healthcare process	Radiation therapy	Patient identification in Radiology department	15	No
[62]	2022	Japan	Prospective analytical study	Healthcare process	Radiation therapy	Anaphylaxis reduction due to contrast media in Radiology	177	Yes
[63]	2023	Germany	Prospective analytical study	Healthcare process	Radiation therapy	Radiation oncology	36	No
[64]	2005	New Zealand	Prospective analytical study	Healthcare process	Medication use process	Neonatal ICU medication use process	72	Yes
[65]	2022	Italy	Prospective analytical study	Healthcare process	Other treatment processes	Dialysis	31	No
[66]	2012	Italy	Prospective analytical study	Healthcare process	Other treatment processes	Drugs prescription and administration in paediatric wards	37	Yes
**[67]**	**2021**	**Morocco**	**Prospective analytical study**	**Healthcare process**	**Infectious diseases, antimicrobials and infection control**	**Drugs prescription and administration during SARS-CoV-2 pandemic**	**12**	**Yes**
[68]	2017	South Korea	Prospective analytical study	Healthcare process	Healthcare support activities	Clinical trials	114	Yes
[69]	2017	USA	Prospective analytical study	Healthcare process	Radiation therapy	Ocular brachytherapy	188	Yes
[70]	2021	The Netherlands	Prospective analytical study	Healthcare process	Other treatment processes	Drug adherence for patients with pheochromocytoma	69	Yes
**[71]**	**2017**	**China**	**Prospective analytical study**	**Healthcare process**	**Infectious diseases, antimicrobials and infection control**	**Prevention of catheter-related infection in ICU**	**25**	**Yes**

**[72]**	**2021**	**China**	**Quality improvement study**	**Healthcare process**	**Infectious diseases, antimicrobials and infection control**	**Prevention of MDROs infection in ICU**	**5**	**Yes**
[73]	2022	China	Prospective analytical study	Healthcare process	Chemotherapy	Chemotherapy in lung cancer	60	Yes
[74]	2020	Canada	Prospective analytical study	Healthcare process	Radiation therapy	Near misses in radiation therapy	107	No
[75]	2022	USA	Prospective analytical study	Healthcare process	Healthcare support activities	Mechanical thrombectomy for stroke	7	Yes
[76]	2023	USA	Quality improvement study	Healthcare process	Blood transfusion	Manual vs automated blood grouping processes	5	Yes
[77]	2013	China	Quality improvement study	Healthcare process	Blood transfusion	Blood transfusion	19	Yes
[78]	2023	China	Quality improvement study	Healthcare process	Testing in clinical laboratory	Laboratory testing (CBC)	6	Yes
[79]	2011	USA	Prospective analytical study	Healthcare process	Medication use process	Drug shortages	8	Yes
[80]	2020	Italy	Quality improvement study	Healthcare process	Healthcare support activities	IVF process	10	Yes
[81]	2020	Italy	Quality improvement study	Healthcare process	Radiation therapy	Radiation oncology	67+27	Yes
[82]	2022	USA	Narrative review	Healthcare process	Other treatment processes	Skin biopsy	1	No
[83]	2017	USA	Quality improvement study	Healthcare process	Other treatment processes	Paediatric anaesthesia	8	Yes
[84]	2023	Sweden	Prospective analytical study	Healthcare process	Healthcare support activities	Drugs supply chain	11	Yes
[85]	2014	Italy	Prospective analytical study	Healthcare process	Radiation therapy	Intracranial stereotactic radiation surgery	116	Yes
[86]	2015	USA	Prospective analytical study	Healthcare process	Radiation therapy	Gynecologic brachytherapy	170	Yes
[87]	2015	USA	Prospective analytical study	Hospital management	Communication and patient handoff	Operating room to ICU handoff	79	No
[88]	2022	Italy	Prospective analytical study	Healthcare process	Other treatment processes	Perioperative management model of oral anticoagulant therapy	17	Yes
[89]	2016	Spain	Quality improvement study	Healthcare process	Healthcare support activities	Patient safety in paediatric emergency care	49	Yes

[90]	2019	Spain	Quality improvement study	Healthcare process	Blood transfusion	Blood transfusion	34	Yes
[91]	2022	Spain	Quality improvement study	Healthcare process	Healthcare support activities	Patient safety in paediatric emergency care	106+110	Yes
[92]	2017	Brazil	Quality improvement study	Healthcare process	Healthcare support activities	Orthotics, prosthetics, and special materials	16	Yes
[93]	2017	Iran	Quality improvement study	Healthcare process	Blood transfusion	Blood transfusion	31	Yes
[94]	2014	Denmark	Prospective analytical study	Healthcare process	Other treatment processes	Obstructed breech delivery	40	No
[95]	2022	Japan	Prospective analytical study	Healthcare process	Radiation therapy	Magnetic resonance-guided radiotherapy	153	No
[96]	2018	Israel	Quality improvement study	Healthcare process	Healthcare support activities	Consultations	44	No
[97]	2014	USA	Prospective analytical study	Hospital management	Treatment process management	Clinical implementation of adaptive radiotherapy	216	Yes
[98]	2021	USA	Prospective analytical study	Healthcare process	Radiation therapy	Prostate brachytherapy	29	No
[99]	2016	Israel	Quality improvement study	Medical equipment/production	None	Ready-to-use diluted potassium chloride solution	13	Yes
[100]	2019	USA	Prospective analytical study	Hospital management	Communication and patient handoff	Documentation discrepancies at transfer out of the pediatric ICU	N/A	No
[101]	2022	Italy	Quality improvement study	Hospital informatization	None	Remote patient telemonitoring	N/A	Yes
[102]	2016	Italy	Prospective analytical study	Healthcare process	Chemotherapy	Subcutaneous vs intravenous chemotherapy	35	No
[103]	2020	USA	Quality improvement study	Healthcare process	Other treatment processes	Analgesia, sedation, and paralysis order set	3	Yes
[104]	2020	Spain	Quality improvement study	Healthcare process	Chemotherapy	Chemotherapy preparation	9	Yes
[105]	2019	USA	Prospective analytical study	Healthcare process	Radiation therapy	Breast radiotherapy	97	No
[106]	2019	United Kingdom	Quality improvement study	Medical equipment/production	None	Pre-prepared standardized concentrations of morphine for paediatric analgesia	5	Yes

[107]	2020	USA	Prospective analytical study	Hospital management	Treatment process management	Initial plan check checklist in radiation treatment	50	No
[108]	2009	United Kingdom	Prospective analytical study	Hospital management	Communication and patient handoff	Communication in the ED	16	No
[109]	2018	Iran	Prospective analytical study	Hospital management	Healthcare support management	Patient journey process in surgery ward	13	No
[110]	2008	USA	Prospective analytical study	Healthcare process	Medication use process	Medication administration	3+6	No
[111]	2017	Italy	Quality improvement study	Hospital informatization	None	Implementation of an electronic witnessing system	293	Yes
[112]	2015	Italy	Quality improvement study	Hospital informatization	None	Implementation of an electronic witnessing system	32	Yes
[113]	2021	USA	Quality improvement study	Healthcare process	Radiation therapy	Brachytherapy	51+105	Yes
[114]	2017	Spain	Prospective analytical study	Healthcare process	Testing in clinical laboratory	Preanalytical process	24+16	Yes
[115]	2022	Spain	Quality improvement study	Healthcare process	Radiation therapy	Radiopharmacy	96	Yes
[116]	2014	USA	Prospective analytical study	Medical equipment/production	None	Universal anaesthesia machine	16	Yes
[117]	2020	USA	Prospective analytical study	Healthcare process	Radiation therapy	Liver radiotherapy	102	Yes
[118]	2020	Ireland	Quality improvement study	Hospital management	Treatment process management	MRI safety in patients with CIEDs	20	Yes
**[119]**	**2021**	**USA**	**Prospective analytical study**	**Healthcare process**	**Infectious diseases, antimicrobials and infection control**	**OPAT**	**13+10**	**Yes**
[120]	2016	Spain	Quality improvement study	Healthcare process	ICU process	Continuous renal replacement therapy	24	Yes
[121]	2020	Iran	Prospective analytical study	Hospital management	Others	Safety management in hospitals	84	No
[122]	2022	Greece	Narrative review	Healthcare process	Radiation therapy	Stereotactic radiation surgery	N/A	Yes
[123]	2016	USA	Prospective analytical study	Healthcare process	Medication use process	Medication administration	35	Yes
[124]	2015	USA	Prospective analytical study	Healthcare process	Radiation therapy	Skin brachytherapy	27	No

[125]	2021	Switzerland	Quality improvement study	Hospital management	Others	Hospital pharmacy relocation	86	Yes
[126]	2022	USA	Quality improvement study	Hospital management	Others	Patient no-show rates reduction	10	Yes
[127]	2022	China	Randomized clinical trial	Healthcare process	Other treatment processes	Craniocerebral injury treatment	N/A	Yes
[128]	2009	United Kingdom	Prospective analytical study	Healthcare process	Medication use process	Intrathecal drug delivery for pain management	50	Yes
[129]	2019	China	Quality improvement study	Healthcare process	Radiation therapy	Helical tomotherapy	122	Yes
[130]	2017	USA	Prospective analytical study	Healthcare process	Surgery	Bilateral same-day cataract surgery process	15	Yes
[131]	2020	The Netherlands	Prospective analytical study	Healthcare process	Radiation therapy	Radiation oncology	142	Yes
[132]	2023	USA	Quality improvement study	Healthcare process	Radiation therapy	Intensity modulated radiation therapy	100	Yes
[133]	2019	United Kingdom	Prospective analytical study	Healthcare process	Healthcare support activities	ADHD management	22	Yes
[134]	2003	USA	Narrative review	Healthcare process	Healthcare process	All	N/A	N/A
[135]	2021	Serbia	Systematic review	Healthcare process	Medication use process	Medication use process	N/A	N/A
[136]	2012	USA	Quality improvement study	Medical equipment/production	None	Smart infusion pump	39+69	Yes
[137]	2016	Iran	Prospective analytical study	Healthcare process	Surgery	Emergency surgical processes	217	Yes
[138]	2015	Brazil	Prospective analytical study	Healthcare process	Radiation therapy	Stereotactic radiation surgery	153+104+131	No
**[139]**	**2021**	**Ethiopia**	**Cross-sectional study**	**Hospital management**	**Healthcare support management**	**Prevention of SARS-CoV-2 transmission in ED**	**22**	**Yes**
[140]	2022	Italy	Quality improvement study	Healthcare process	Other treatment processes	Bone marrow donation	28	Yes
[141]	2011	USA	Prospective analytical study	Healthcare process	Radiation therapy	Radiation oncology	17	Yes
[142]	2013	The Netherlands	Prospective analytical study	Healthcare process	Other treatment processes	Supplemental oxygen therapy in preterm infants	134	Yes
[143]	2022	Belgium	Quality improvement study	Healthcare process	Testing in clinical laboratory	Preanalytical process	17	Yes

[144]	2018	Spain	Prospective analytical study	Healthcare process	ICU process	ICU process	101	Yes
[145]	2016	Spain	Prospective analytical study	Healthcare process	ICU process	Suctioning by orotracheal tube	32	Yes
[146]	2015	Italy	Prospective analytical study	Healthcare process	Radiation therapy	Stereotactic radiation surgery	19	Yes
[147]	2020	USA	Quality improvement study	Healthcare process	Radiation therapy	Radiation oncology	33	Yes
[148]	2011	Belgium	Prospective analytical study	Healthcare process	Radiation therapy	Radiation oncology	86	Yes
[149]	2016	Saudi Arabia	Prospective analytical study	Healthcare process	Radiation therapy	Brachytherapy	40	Yes
[150]	2013	USA	Prospective analytical study	Healthcare process	Chemotherapy	Pediatric oral cancer chemotherapy	69	Yes
[151]	2022	Germany	Prospective analytical study	Healthcare process	Chemotherapy	Intravenous cancer chemotherapy	52	Yes
[152]	2004	USA	Prospective analytical study	Healthcare process	Other treatment processes	Extracorporeal circuits for cardiopulmonary bypass	77	No
[153]	2011	USA	Prospective analytical study	Healthcare process	Chemotherapy	Oral chemotherapy	325	Yes
[154]	2006	USA	Prospective analytical study	Medical equipment/production	None	Smart infusion pump	200	Yes
[155]	2017	USA	Prospective analytical study	Healthcare process	Radiation therapy	Gamma Knife radiosurgery	86	Yes
**[156]**	**2021**	**China**	**Prospective analytical study**	**Hospital management**	**Healthcare support management**	**Prevention of MDROs infections**	**7**	**Yes**
[157]	2019	USA	Prospective analytical study	Healthcare process	Radiation therapy	Radiotherapy treatment delay	25	No
[158]	2015	USA	Prospective analytical study	Healthcare process	Radiation therapy	Stereotactic radiation surgery	63	No
[159]	2016	USA	Prospective analytical study	Healthcare process	Radiation therapy	Brachytherapy	174	Yes
[160]	2014	USA	Prospective analytical study	Healthcare process	Radiation therapy	Stereotactic radiation surgery	99	No
[161]	2018	Taiwan	Prospective analytical study	Hospital management	Healthcare support management	Infant abduction prevention	16	No
[162]	2019	China	Quality improvement study	Hospital management	Treatment process management	Prevention of posture syndrome of thyroid surgery	7	Yes
[163]	2021	China	Prospective analytical study	Hospital management	Communication and patient handoff	Intrahospital transport	64	Yes

References

[1] Liu H, Zhang L, Ping Y, et al. Failure mode and effects analysis for proactive healthcare risk evaluation: A systematic literature review. J Eval Clin Pract. 2020;26(4):1320–1337.

[2] Abbassi A, Ben Cheikh Brahim A, Ouahchi Z. Failure mode and effect analysis applied to improve the medication management process in a pharmacy of a teaching hospital and a proposal for a simplified rating system. Eur J Hosp Pharm. 2023;30(e1):e55–e60.

[3] Ahmed K, Khan N, Khan MS, et al. Development and content validation of a surgical safety checklist for operating theatres that use robotic technology. BJU Int. 2013;111(7):1161–1174.

[4] Ahmed S, Bossenberger T, Nalichowski A, et al. A bi-institutional multi-disciplinary failure mode and effects analysis (FMEA) for a Co-60 based total body irradiation technique. Radiat Oncol. 2021;16(1):224.

[5] Al Tehewy M, El Hosseini M, Habil I, et al. A proactive risk management using failure mode and effects analysis for infusion therapy in a tertiary hospital intensive care unit in Egypt. Acta Medica Mediterr. 2015;31:195–200.

[6] Alamry A, Al Owais SM, Marini AM, et al. Application of Failure Mode Effect Analysis to Improve the Care of Septic Patients Admitted Through the Emergency Department. J Patient Saf. 2017;13(2):76–81.

[7] Alimohammadzadeh K, Bahadori M, Jahangir T, et al. Assessing the Common Medical Errors in a Children's Hospital NICU Using Failure Mode and Effects Analysis (EMEA). Trauma Mon [Internet]. 2017 [cited 2024 Sep 5];In Press(In Press).

[8] Almeida J, Araujo CAS, De Aguiar Roza B, et al. Risk Analysis of the Organ Donation-Transplantation Process in Brazil. Transplant Proc. 2021;53(2):607–611.

[9] Aly NAEM, El-Shanawany SM, Maher Ghoneim TA. Using Failure Mode and Effects Analysis in Blood Administration Process in Surgical Care Units: New Categories of Errors. Qual Manag Health Care. 2020;29(4):242–252.

[10] Anjalee JAL, Rutter V, Samaranayake NR. Application of Failure Mode and Effect Analysis (FMEA) to improve medication safety: a systematic review. Postgrad Med J. 2021;97(1145):168–174.

[11] Aranaz-Andrés JM, Bermejo-Vicedo T, Muñoz-Ojeda I. Failure mode and effects analysis applied to the administration of liquid medication by oral syringes.

[12] Arenas Jiménez MD, Ferre G, Álvarez-Ude F. Strategies to increase patient safety in haemodialysis: Application of the modal analysis system of errors and effects (FEMA system). Nefrol Engl Ed. 2017;37(6):608–621.

[13] Arenas Villafranca JJ, Sánchez AG, Guindo MN, et al. Using failure mode and effects analysis to improve the safety of neonatal parenteral nutrition. Am J Health Syst Pharm. 2014;71(14):1210–1218.

[14] Asefzadeh S, Yarmohammadian MH, Nikpey A, et al. Clinical Risk Assessment in Intensive Care Unit. Int J Prev Med. 2013;4(5).

[15] Evaluating the application of failure mode and effects analysis technique in hospital wards: a systematic review. J Inj Violence Res [Internet]. 2017 [cited 2024 Sep 5];9(1).

[16] Franklin BD, Shebl NA, Barber N. Failure mode and effects analysis: too little for too much? BMJ Qual Saf. 2012;21(7):607–611.

[17] Ashley L, Dexter R, Marshall F, et al. Improving the Safety of Chemotherapy Administration: An Oncology Nurse-Led Failure Mode and Effects Analysis. Oncol Nurs Forum. 2011;38(6):E436–E444.

[18] Baehr A, Oertel M, Kröger K, et al. Implementing a new scale for failure mode and effects analysis (FMEA) for risk analysis in a radiation oncology department. Strahlenther Onkol. 2020;196(12):1128–1134.

[19] Bhirich N, Chefchaouni AC, Medkouri SE, et al. Risk assessment of personnel exposure in a central cytotoxic preparation unit using the FMECA method. J Oncol Pharm Pract. 2023;29(8):1884–1892.

[20] Bonfant G, Belfanti P, Paternoster G, et al. Clinical risk analysis with failure mode and effect analysis (FMEA) model in a dialysis unit. 2010;

[21] Bright M, Foster RD, Hampton CJ, et al. Failure modes and effects analysis for surface-guided DIBH breast radiotherapy. J Appl Clin Med Phys. 2022;23(4):e13541.

[22] Buja A, De Luca G, Ottolitri K, et al. Using Failure Mode, Effect and Criticality Analysis to improve safety in the cancer treatment prescription and administration process. J Pharm Policy Pract. 2023;16(1):9.

[23] Canepa P, De Leo C, Casciano I, et al. Failure modes and effects analysis for testicular sperm extraction management process. Andrologia [Internet]. 2020 [cited 2024 Sep 5];52(3).

[24] Cantone MC, Ciocca M, Dionisi F, et al. Application of failure mode and effects analysis to treatment planning in scanned proton beam radiotherapy. Radiat Oncol. 2013;8(1):127.

[25] Chiereghin A, Squillace L, Pizzi L, et al. Applying the healthcare failure mode and effects analysis approach to improve the quality of an organised colorectal cancer screening programme. J Med Screen. 2024;31(2):70–77.

[26] Chiozza ML, Ponzetti C. FMEA: A model for reducing medical errors. Clin Chim Acta. 2009;404(1):75–78.

[27] Culebras Diaz AM, Gordo C, Mateo R, et al. Associations of wrong surgery with other critical healthcare quality and patient safety challenges: a cross-sectional nationwide study of 100 general hospitals in Spain. Surg Today. 2023;53(2):269–273.

[28] Daniels LM, Barreto JN, Kuth JC, et al. Failure mode and effects analysis to reduce risk of anticoagulation levels above the target range during concurrent antimicrobial therapy. Am J Health Syst Pharm. 2015;72(14):1195–1203.

[29] Davis NR, Doughty CB, Kerr T, et al. Rapidly building surge capacity within a pandemic response using simulation-based clinical systems testing. BMJ Simul Technol Enhanc Learn. 2021;7(5):304–310.

[30] Day S, Dalto J, Fox J, et al. Utilization of Failure Mode Effects Analysis in Trauma Patient Registration. Qual Manag Health Care. 2007;16(4):342–348.

[31] Day S, Dalto J, Fox J, et al. Failure mode and effects analysis as a performance improvement tool in trauma. J Trauma Nurs. 2006;13(3):111–117.

[32] Dehnavieh R, Ebrahimipour H, Molavi-Taleghani Y, et al. Proactive Risk Assessment of Blood Transfusion Process, in Pediatric Emergency, Using the Health Care Failure Mode and Effects Analysis (HFMEA). Glob J Health Sci. 2014;7(1):p322.

[33] Denny DS, Allen DK, Worthington N, et al. The Use of Failure Mode and Effect Analysis in a Radiation Oncology Setting: The Cancer Treatment Centers of America Experience. J Healthc Qual. 2014;36(1):18–28.

[34] Eilithy MH, Alsamani O, Salah H, et al. Challenges experienced during pharmacy automation and robotics implementation in JCI accredited hospital in the Arabian Gulf area: FMEA analysis-qualitative approach. Saudi Pharm J. 2023;31(9):101725.

[35] Faught JT, Balter PA, Johnson JL, et al. An FMEA evaluation of intensity modulated radiation therapy dose delivery failures at tolerance criteria levels. Med Phys. 2017;44(11):5575–5583.

[36] Feemster AA, Augustino M, Duncan R, et al. Use of failure modes and effects analysis to mitigate potential risks prior to implementation of an intravenous compounding technology. Am J Health Syst Pharm. 2021;78(14):1323–1329.

[37] Flegar-Meštrić Z, Perkov S, Radeljak A, et al. Risk analysis of the preanalytical process based on quality indicators data. Clin Chem Lab Med CCLM [Internet]. 2017 [cited 2024 Sep 5];55(3).

[38] Ford EC, Gaudette R, Myers L, et al. Evaluation of Safety in a Radiation Oncology Setting Using Failure Mode and Effects Analysis. Int J Radiat Oncol. 2009;74(3):852–858.

[39] Frewen H, Brown E, Jenkins M, et al. Failure mode and effects analysis in a paperless radiotherapy department. J Med Imaging Radiat Oncol. 2018;62(5):707–715.

[40] Funk KH, Bauer JD, Doolen TL, et al. Use of Modeling to Identify Vulnerabilities to Human Error in Laparoscopy. J Minim Invasive Gynecol. 2010;17(3):311–320.

[41] Giardina M, Cantone MC, Tomarchio E, et al. A Review of Healthcare Failure Mode and Effects Analysis (HFMEA) in Radiotherapy. Health Phys. 2016;111(4):317–326.

[42] Gilmore MDF, Rowbottom CG. Evaluation of failure modes and effect analysis for routine risk assessment of lung radiotherapy at a UK center. J Appl Clin Med Phys. 2021;22(5):36–47.

[43] Gray T, Antolak A, Ahmed S, et al. Implementing failure mode and effect analysis to improve the safety of volumetric modulated arc therapy for total body irradiation. Med Phys. 2023;50(7):4092–4104.

[44] Guédon AC, Rakers TJ, Wauben LSGL, et al. Just-in-time delivery of sterilised surgical instruments. BMJ Innov. 2016;2(2):58–64.

[45] Guédon ACP, Wauben LSGL, Van Der Eijk AC, et al. Where are my instruments? Hazards in delivery of surgical instruments. Surg Endosc. 2016;30(7):2728–2735.

[46] Soler A, Alvarez L, Mira A, et al. Analytical performance assessment and improvement by means of the Failure mode and effect analysis (FMEA). Biochem Medica. 2020;30(2):250–256.

[47] Gurses AP, Dietz AS, Nowakowski E, et al. Human factors–based risk analysis to improve the safety of doffing enhanced personal protective equipment. Infect Control Hosp Epidemiol. 2019;40(2):178–186.

[48] Hagle M, Snyder K, Janicek K, et al. Using a Patient Safety Analysis to Guide Infusion Therapy for Patients With COVID-19. J Infus Nurs. 2021;44(5):259–267.

[49] Han TH, Kim MJ, Kim S, et al. The role of failure modes and effects analysis in showing the benefits of automation in the blood bank. Transfusion (Paris). 2013;53(5):1077–1082.

[50] Haroun A, Al- Ruzzieh M, Hussien N, et al. Using Failure Mode and Effects Analysis in Improving Nursing Blood Sampling at an International Specialized Cancer Center. Asian Pac J Cancer Prev. 2021;22(4):1247–1254.

[51] He H, Wang F, Zhou C, et al. Optimization of the emergency endoscopy process for patients with esophagogastric variceal bleeding using failure mode and effect analysis.

[52] Ho CC, Liao C-J. The use of failure mode and effects analysis to construct an effective disposal and prevention mechanism for infectious hospital waste. Waste Manag. 2011;31(12):2631–2637.

[53] Huang S-F, Cheng H-W, Tsai J-T, et al. Failure mode and effects analysis for errors detected during pretreatment physics plan and chart review in external beam radiotherapy. Ther Radiol Oncol. 2022;6:11–11.

[54] Huergo Fernández A, Amor Martín P, Fernández Cadenas F. Propofol sedation implementation: utility of the failure mode and effect analysis. Patient focused quality and safety. Rev Esp Enfermedades Dig [Internet]. 2017 [cited 2024 Sep 5];109.

[55] Huq MS, Fraass BA, Dunscombe PB, et al. A Method for Evaluating Quality Assurance Needs in Radiation Therapy. Int J Radiat Oncol. 2008;71(1):S170–S173.

[56] Islam NM, Wadi-Ramahi SJ, Lalonde R, et al. A novel approach to infectious disease control and radiotherapy risk management. Med Phys. 2023;50(5):2683–2694.

[57] Jafarzadeh Ghoushchi S, Dorosti S, Ab Rahman MN, et al. Theory-Based Failure Modes and Effect Analysis for Medication Errors. Chen C-H, editor. J Healthc Eng. 2021;2021:1–14.

[58] Jones RT, Handsfield L, Read PW, et al. Safety and feasibility of STAT RAD: Improvement of a novel rapid tomotherapy-based radiation therapy workflow by failure mode and effects analysis. Pract Radiat Oncol. 2015;5(2):106–112.

[59] Kisling K, Johnson JL, Simonds H, et al. A risk assessment of automated treatment planning and recommendations for clinical deployment. Med Phys. 2019;46(6):2567–2574.

[60] Klüter S, Schrenk O, Renkamp CK, et al. A practical implementation of risk management for the clinical introduction of online adaptive Magnetic Resonance-guided radiotherapy. Phys Imaging Radiat Oncol. 2021;17:53–57.

[61] Kobo-Greenhut A, Sharlin O, Adler Y, et al. Algorithmic prediction of failure modes in healthcare. Int J Qual Health Care. 2021;33(1):mzaa151.

[62] Koike D, Yamakami J, Miyashita T, et al. Combining failure modes and effects analysis and cause–effect analysis: a novel method of risk analysis to reduce anaphylaxis due to contrast media. Int J Qual Health Care [Internet]. 2022 [cited 2024 Sep 5];34(1).

[63] Kornek D, Menichelli D, Leske J, et al. Development and clinical implementation of a digital system for risk assessments for radiation therapy. Z Für Med Phys. 2024;34(3):371–383.

[64] Kunac DL, Reith DM. Identification of Priorities for Medication Safety in Neonatal Intensive Care: Drug Saf. 2005;28(3):251–261.

[65] La Russa R, Fazio V, Ferrara M, et al. Proactive Risk Assessment Through Failure Mode and Effect Analysis (FMEA) for Haemodialysis Facilities: A Pilot Project. Front Public Health. 2022;10:823680.

[66] Lago P, Bizzarri G, Scalzotto F, et al. Use of FMEA analysis to reduce risk of errors in prescribing and administering drugs in paediatric wards: a quality improvement report. BMJ Open. 2012;2(6):e001249.

[67] E. E. V. Latt, Y. Moutaouakkil, Z. Qriouet, et al. Failure Mode and Effect Analysis: A Technique to Prevent the Risk of SARS-COV-2 Infection in A Retrocession Unit. Indian J Forensic Med Toxicol [Internet]. 2021 [cited 2024 Sep 5];15(3).

[68] Lee H, Lee H, Baik J, et al. Failure mode and effects analysis drastically reduced potential risks in clinical trial conduct. Drug Des Devel Ther. 2017;Volume 11:3035–3043.

[69] Lee YC, Kim Y, Huynh JW-Y, et al. Failure modes and effects analysis for ocular brachytherapy. Brachytherapy. 2017;16(6):1265–1279.

[70] Leeftink AG, Visser J, De Laat JM, et al. Reducing failures in daily medical practice: Healthcare failure mode and effect analysis combined with computer simulation. Ergonomics. 2021;64(10):1322–1332.

[71] Li X, He M, Wang H. Application of failure mode and effect analysis in managing catheter-related blood stream infection in intensive care unit. Medicine (Baltimore). 2017;96(51):e9339.

[72] Lin L, Wang R, Chen T, et al. Failure mode and effects analysis on the control effect of multi-drug-resistant bacteria in ICU patients.

[73] Lin S, Wang N, Ren B, et al. Use of Failure Mode and Effects Analysis (FMEA) for Risk Analysis of Drug Use in Patients with Lung Cancer. Int J Environ Res Public Health. 2022;19(23):15428.

[74] Liszewski B. A prioritization framework for the analysis of near misses in radiation oncology. Tech Innov Patient Support Radiat Oncol. 2020;14:36–42.

[75] Liu Y, Gebrezgiabhier D, Reddy AS, et al. Failure modes and effects analysis of mechanical thrombectomy for stroke discovered in human brains. J Neurosurg. 2022;136(1):197–204.

[76] Lou SS, Dewey MM, Bollini ML, et al. Reducing perioperative red blood cell unit issue orders, returns, and waste using failure modes and effects analysis. Transfusion (Paris). 2023;63(4):755–762.

[77] Lu Y, Teng F, Zhou J, et al. Failure mode and effect analysis in blood transfusion: a proactive tool to reduce risks. Transfusion (Paris). 2013;53(12):3080–3087.

[78] Lv S, Sun Y, Zhang J, et al. Application of the FMEA Method in Improving the Quality Management of Emergency Complete Blood Count Testing. Lab Med. 2023;54(6):574–581.

[79] Elyse AM, Erin RF, Linda ST. Drug Shortages: Process for Evaluating Impact on Patient Safety. Hosp Pharm. 2011;46(12):943–951.

[80] Maggiulli R, Giancani A, Fabozzi G, et al. Assessment and management of the risk of SARS-CoV-2 infection in an IVF laboratory. Reprod Biomed Online. 2020;41(3):385–394.

[81] Mancosu P, Signori C, Clerici E, et al. Critical Re-Evaluation of a Failure Mode Effect Analysis in a Radiation Therapy Department After 10 Years. Pract Radiat Oncol. 2021;11(3):e329–e338.

[82] Marsch A, Khodosh R, Porter M, et al. Implementing patient safety and quality improvement in dermatology. Part 1: Patient safety science. J Am Acad Dermatol. 2023;89(4):641–654.

[83] Martin LD, Grigg EB, Verma S, et al. Outcomes of a Failure Mode and Effects Analysis for medication errors in pediatric anesthesia. Cravero J, editor. Pediatr Anesth. 2017;27(6):571–580.

[84] Martínez CS, Amery L, De Paoli G, et al. Examination of the Protein Drug Supply Chain in a Swedish University Hospital: Focus on Handling Risks and Mitigation Measures. J Pharm Sci. 2023;112(11):2799–2810.

[85] Masini L, Donis L, Loi G, et al. Application of failure mode and effects analysis to intracranial stereotactic radiation surgery by linear accelerator. Pract Radiat Oncol. 2014;4(6):392–397.

[86] Mayadev J, Dieterich S, Harse R, et al. A failure modes and effects analysis study for gynecologic high-dose-rate brachytherapy. Brachytherapy. 2015;14(6):866–875.

[87] McElroy LM, Khorzad R, Nannicelli AP, et al. Failure mode and effects analysis: a comparison of two common risk prioritisation methods. BMJ Qual Saf. 2016;25(5):329–336.

[88] Micheletta F, Ferrara M, Bertozzi G, et al. Proactive Risk Assessment through Failure Mode and Effect Analysis (FMEA) for Perioperative Management Model of Oral Anticoagulant Therapy: A Pilot Project. Int J Environ Res Public Health. 2022;19(24):16430.

[89] Mojica E, Izarzugaza E, Gonzalez M, et al. Elaboration of a risk map in a paediatric Emergency Department of a teaching hospital. Emerg Med J. 2016;33(10):684–689.

[90] Mora A, Ayala L, Bielza R, et al. Improving safety in blood transfusion using failure mode and effect analysis. Transfusion (Paris). 2019;59(2):516–523.

[91] Mora-Capín A, Ignacio-Cerro C, Díaz-Redondo A, et al. Impact of risk mapping as a strategy for monitoring and improving patient safety in paediatric emergency care. An Pediatría Engl Ed. 2022;97(4):229–236.

[92] Moraes CDS, Rabin EG, Viégas K. Assessment of the care process with orthotics, prosthetics and special materials. Rev Bras Enferm. 2018;71(3):1099–1105.

[93] Najafpour Z, Hasoumi M, Behzadi F, et al. Preventing blood transfusion failures: FMEA, an effective assessment method. BMC Health Serv Res. 2017;17(1):453.

[94] Nielsen DS, Dieckmann P, Mohr M, et al. Augmenting Health Care Failure Modes and Effects Analysis With Simulation. Simul Healthc J Soc Simul Healthc. 2014;9(1):48–55.

[95] Nishioka S, Okamoto H, Chiba T, et al. Identifying risk characteristics using failure mode and effect analysis for risk management in online magnetic resonance-guided adaptive radiation therapy. Phys Imaging Radiat Oncol. 2022;23:1–7.

[96] Niv Y, Itskoviz D, Cohen M, et al. The Utility of Failure Modes and Effects Analysis of Consultations in a Tertiary, Academic, Medical Center. Qual Manag Health Care. 2018;27(2):69–73.

[97] Noel CE, Santanam L, Parikh PJ, et al. Process-based quality management for clinical implementation of adaptive radiotherapy. Med Phys. 2014;41(8Part1):081717.

[98] Nunez DA, Trager M, Beaudry J, et al. Quantifying clinical severity of physics errors in high-dose rate prostate brachytherapy using simulations. Brachytherapy. 2021;20(5):1062–1069.

[99] Ofek F, Magnezi R, Kurzweil Y, et al. Introducing a change in hospital policy using FMEA methodology as a tool to reduce patient hazards. Isr J Health Policy Res. 2016;5(1):30.

[100] Orenstein EW, Ferro DF, Bonafide CP, et al. Hidden health IT hazards: a qualitative analysis of clinically meaningful documentation discrepancies at transfer out of the pediatric intensive care unit. JAMIA Open. 2019;2(3):392–398.

[101] Parretti C, Tartaglia R, La Regina M, et al. Improved FMEA Methods for Proactive Health Care Risk Assessment of the Effectiveness and Efficiency of COVID-19 Remote Patient Telemonitoring. Am J Med Qual. 2022;37(6):535–544.

[102] Walzer S, Ponzetti C, Canciani M, et al. Administrative risk quantification of subcutaneous and intravenous therapies in Italian centers utilizing the Failure Mode and Effects Analysis approach. Clin Outcomes Res. 2016;Volume 8:353–359.

[103] Procaccini D, Rapaport R, Petty B, et al. Design and Implementation of an Analgesia, Sedation, and Paralysis Order Set to Enhance Compliance of pro re nata Medication Orders with Joint Commission Medication Management Standards in a Pediatric ICU. Jt Comm J Qual Patient Saf. 2020;46(12):706–714.

[104] Pueyo-López C, Sánchez-Cuervo M, Vélez-Díaz-Pallarés M, et al. Healthcare failure mode and effect analysis in the chemotherapy preparation process. J Oncol Pharm Pract. 2021;27(7):1588–1595.

[105] Rash D, Hoffman D, Manger R, et al. Risk analysis of electronic intraoperative radiation therapy for breast cancer. Brachytherapy. 2019;18(3):271–276.

[106] Rashed AN, Whittlesea C, Davies C, et al. Standardised concentrations of morphine infusions for nurse/patient-controlled analgesia use in children. BMC Anesthesiol. 2019;19(1):26.

[107] Rassiah P, Su FF, Huang YJ, et al. Using failure mode and effects analysis (FMEA) to generate an initial plan check checklist for improved safety in radiation treatment. J Appl Clin Med Phys. 2020;21(8):83–91.

[108] Redfern E, Brown R, Vincent CA. Identifying vulnerabilities in communication in the emergency department. Emerg Med J. 2009;26(9):653–657.

[109] Rezaei F, Yarmohammadian M, Haghshenas A, et al. Revised risk priority number in failure mode and effects analysis model from the perspective of healthcare system. Int J Prev Med. 2018;9(1):7.

[110] Riehle MA, Bergeron D, Hyrkäs K. Improving process while changing practice: FMEA and medication administration. Nurs Manag (Harrow). 2008;28–33.

[111] Rienzi L, Bariani F, Dalla Zorza M, et al. Comprehensive protocol of traceability during IVF: the result of a multicentre failure mode and effect analysis. Hum Reprod. 2017;32(8):1612–1620.

[112] Rienzi L, Bariani F, Dalla Zorza M, et al. Failure mode and effects analysis of witnessing protocols for ensuring traceability during IVF. Reprod Biomed Online. 2015;31(4):516–522.

[113] Roles SA, Hepel JT, Leonard KL, et al. Quantifying risk using FMEA: An alternate approach to AAPM TG-100 for scoring failures and evaluating clinical workflow. Brachytherapy. 2021;20(4):922–935.

[114] Romero A, Gómez-Salgado J, Romero-Arana A, et al. Utilization of a healthcare failure mode and effects analysis to identify error sources in the preanalytical phase in two tertiary hospital laboratories. Biochem Medica. 2018;28(2):020713.

[115] Romero-Zayas I, Campos Añón F, Santos Virosta M, et al. Implementation of the failure modes and effects analysis in a Hospital Radiopharmacy Unit. Rev Esp Med Nucl E Imagen Mol Engl Ed. 2022;41(5):300–310.

[116] Rosen MA, Sampson JB, Jackson EV, et al. Failure mode and effects analysis of the universal anaesthesia machine in two tertiary care hospitals in Sierra Leone. Br J Anaesth. 2014;113(3):410–415.

[117] Rusu I, Thomas TO, Roeske JC, et al. Failure mode and effects analysis of linac-based liver stereotactic body radiotherapy. Med Phys. 2020;47(3):937–947.

[118] Ryan JW, Murray AS, Gilligan PJ, et al. MRI safety management in patients with cardiac implantable electronic devices: Utilizing failure mode and effects analysis for risk optimization. Int J Qual Health Care. 2020;32(7):431–437.

[119] Sadler ED, Avdic E, Cosgrove SE, et al. Failure modes and effects analysis to improve transitions of care in patients discharged on outpatient parenteral antimicrobial therapy. Am J Health Syst Pharm. 2021;78(13):1223–1232.

[120] Sanchez-Izquierdo-Riera JA, Molano-Alvarez E, Saez-de La Fuente I, et al. Safety Management of a Clinical Process Using Failure Mode and Effect Analysis: Continuous Renal Replacement Therapies in Intensive Care Unit Patients. ASAIO J. 2016;62(1):74–79.

[121] Saranjam B, Naghizadeh L, Rahimi E, et al. Assessment of Health and Safety Hazards in Hospitals using five methods and comparing the results with the FMEA method.

[122] Sarchosoglou AA, Papavasileiou P, Bakas A, et al. Failure modes in stereotactic radiosurgery. A narrative review. Radiography. 2022;28(4):999–1009.

[123] Saulino MF, Patel T, Fisher SP. The Application of Failure Modes and Effects Analysis Methodology to Intrathecal Drug Delivery for Pain Management. Neuromodulation Technol Neural Interface. 2017;20(2):177–186.

[124] Sayler E, Eldredge-Hindy H, Dinome J, et al. Clinical implementation and failure mode and effects analysis of HDR skin brachytherapy using Valencia and Leipzig surface applicators. Brachytherapy. 2015;14(2):293–299.

[125] Schumacher L, Dobrinas M, Krähenbühl S, et al. Using risk analysis to anticipate and mitigate failures during a hospital pharmacy relocation. Eur J Hosp Pharm. 2021;28(e1):e164–e170.

[126] Set KK, Bailey J, Kumar G. Reduction of No-Show Rate for New Patients in a Pediatric Neurology Clinic. Jt Comm J Qual Patient Saf. 2022;48(12):674–681.

[127] Shao X-L, Wang Y-Z, Chen X-H, et al. Impact of failure mode and effects analysis-based emergency management on the effectiveness of craniocerebral injury treatment. World J Clin Cases. 2022;10(2):554–562.

[128] Shebl NA, Franklin BD, Barber N. Is Failure Mode and Effect Analysis Reliable? J Patient Saf. 2009;5(2):86–94.

[129] Shen J, Wang X, Deng D, et al. Evaluation and improvement the safety of total marrow irradiation with helical tomotherapy using repeat failure mode and effects analysis. Radiat Oncol. 2019;14(1):238.

[130] Shorstein NH, Lucido C, Carolan J, et al. Failure Modes and Effects Analysis of bilateral same-day cataract surgery. J Cataract Refract Surg. 2017;43(3):318–323.

[131] Silvis-Cividjian N, Verbakel W, Admiraal M. Using a systems-theoretic approach to analyze safety in radiation therapy-first steps and lessons learned. Saf Sci. 2020;122:104519.

[132] Simiele E, Han B, Skinner L, et al. Mitigation of Intensity Modulated Radiation Therapy and Stereotactic Body Radiation Therapy Treatment Planning Errors on the Novel RefleXion X1 System Using Failure Mode and Effect Analysis Within Six Sigma Framework. Adv Radiat Oncol. 2023;8(5):101186.

[133] Simsekler MCE, Kaya GK, Ward JR, et al. Evaluating inputs of failure modes and effects analysis in identifying patient safety risks. Int J Health Care Qual Assur. 2019;32(1):191–207.

[134] Spath PL. Using failure mode and effects analysis to improve patient safety. AORN J. 2003;78(1):15–37.

[135] Stojkovic T, Marinkovic V, Manser T. Using Prospective Risk Analysis Tools to Improve Safety in Pharmacy Settings: A Systematic Review and Critical Appraisal. J Patient Saf. 2021;17(6):e515–e523.

[136] Syroid N, Liu D, Albert R, et al. Graphical User Interface Simplifies Infusion Pump Programming and Enhances the Ability to Detect Pump-Related Faults. Anesth Analg. 2012;115(5):1087–1097.

[137] Taleghani YM, Rezaei F, Sheikhbardsiri H. Risk assessment of the emergency processes: Healthcare failure mode and effect analysis. World J Emerg Med. 2016;7(2):97.

[138] Teixeira FC, De Almeida CE, Saiful Huq M. Failure mode and effects analysis based risk profile assessment for stereotactic radiosurgery programs at three cancer centers in Brazil: FMEA risk profile assessment for SRS in Brazil. Med Phys. 2015;43(1):171–178.

[139] Teklewold B, Anteneh D, Kebede D, et al. Use of Failure Mode and Effect Analysis to Reduce Admission of Asymptomatic COVID-19 Patients to the Adult Emergency Department: An Institutional Experience. Risk Manag Healthc Policy. 2021;Volume 14:273–282.

[140] Teofili L, Bianchi M, Valentini CG, et al. Validation plan of bone marrow collection, processing and distribution using the failure mode and effect analysis methodology: a technical report. Cytotherapy. 2022;24(3):356–364.

[141] Thornton E, Brook OR, Mendiratta-Lala M, et al. Application of Failure Mode and Effect Analysis in a Radiology Department. RadioGraphics. 2011;31(1):281–293.

[142] Eijk ACVD, Rook D, Dankelman J, et al. Defining Hazards of Supplemental Oxygen Therapy in Neonatology Using the FMEA Tool. MCN Am J Matern Nurs. 2013;38(4):221–228.

[143] Hoof VV, Bench S, Soto AB, et al. Failure Mode and Effects Analysis (FMEA) at the preanalytical phase for POCT blood gas analysis: proposal for a shared proactive risk analysis model. Clin Chem Lab Med CCLM. 2022;60(8):1186–1201.

[144] Vázquez-Valencia A, Santiago-Sáez A, Perea-Pérez B, et al. Usefulness of failure mode and effects analysis for improving mobilization safety in critically ill patients: Colomb J Anesthesiol. 2018;46(1):3–10.

[145] Vázquez-Valencia A, Santiago-Sáez A, Perea-Pérez B, et al. Utility of Failure Mode and Effect Analysis to Improve Safety in Suctioning by Orotracheal Tube. J Perianesth Nurs. 2017;32(1):28–37.

[146] Veronese I, De Martin E, Martinotti AS, et al. Multi-institutional application of Failure Mode and Effects Analysis (FMEA) to CyberKnife Stereotactic Body Radiation Therapy (SBRT). Radiat Oncol. 2015;10(1):132.

[147] Viscariello N, Evans S, Parker S, et al. A multi-institutional assessment of COVID-19-related risk in radiation oncology. Radiother Oncol. 2020;153:296–302.

[148] Vlayen A. Evaluation of Time- and Cost-Saving Modifications of HFMEA: An Experimental Approach in Radiotherapy. 2011;7(3).

[149] Wadi-Ramahi S, Alnajjar W, Mahmood R, et al. Failure modes and effects analysis in image-guided high-dose-rate brachytherapy: Quality control optimization to reduce errors in treatment volume. Brachytherapy. 2016;15(5):669–678.

[150] Walsh KE, Mazor KM, Roblin D, et al. Multisite Parent-Centered Risk Assessment to Reduce Pediatric Oral Chemotherapy Errors. J Oncol Pract. 2013;9(1):e1–e7.

[151] Weber L, Schulze I, Jaehde U. Using failure mode and Effects Analysis to increase patient safety in cancer chemotherapy. Res Soc Adm Pharm. 2022;18(8):3386–3393.

[152] Wehrli-Veit M, Riley JB, Austin JW. A Failure Mode Effect Analysis on Extracorporeal Circuits for Cardiopulmonary Bypass. J Extracorpor Technol. 2004;36(4):351–357.

[153] Weingart SN, Spencer J, Buia S, et al. Medication Safety of Five Oral Chemotherapies: A Proactive Risk Assessment. J Oncol Pract. 2011;7(1):2–6.

[154] Wetterneck TB, Skibinski KA, Roberts TL, et al. Using failure mode and effects analysis to plan implementation of smart i.v. pump technology. Am J Health Syst Pharm. 2006;63(16):1528–1538.

[155] Xu AY, Bhatnagar J, Bednarz G, et al. Failure modes and effects analysis (FMEA) for Gamma Knife radiosurgery. J Appl Clin Med Phys. 2017;18(6):152–168.

[156] Xu Y, Wang W, Li Z, et al. Effects of healthcare failure mode and effect analysis on the prevention of multi-drug resistant organisms infections in oral and maxillofacial surgery.

[157] Xu Z, Lee S, Albani D, et al. Evaluating radiotherapy treatment delay using Failure Mode and Effects Analysis (FMEA). Radiother Oncol. 2019;137:102–109.

[158] Yang F, Cao N, Young L, et al. Validating FMEA output against incident learning data: A study in stereotactic body radiation therapy: Validating FMEA output against incident learning data. Med Phys. 2015;42(6Part1):2777–2785.

[159] Younge KC, Lee C, Moran JM, et al. Failure mode and effects analysis in a dual-product microsphere brachytherapy environment. Pract Radiat Oncol. 2016;6(6):e299–e306.

[160] Younge KC, Wang Y, Thompson J, et al. Practical Implementation of Failure Mode and Effects Analysis for Safety and Efficiency in Stereotactic Radiosurgery. Int J Radiat Oncol. 2015;91(5):1003–1008.

[161] Yu S-H, Su E, Chen Y-T. Data-Driven Approach to Improving the Risk Assessment Process of Medical Failures. Int J Environ Res Public Health. 2018;15(10):2069.

[162] Zhang L, Zeng L, Yan Y, et al. Application of the healthcare failure mode and effects analysis system to reduce the incidence of posture syndrome of thyroid surgery. Medicine (Baltimore). 2019;98(51):e18309.

[163] Zhang W, Lv J, Zhao J, et al. Proactive risk assessment of intrahospital transport of critically ill patients from emergency department to intensive care unit in a teaching hospital and its implications. J Clin Nurs. 2022;31(17–18):2539–2552.
